# Neuromechanism Study of Insect–Machine Interface: Flight Control by Neural Electrical Stimulation

**DOI:** 10.1371/journal.pone.0113012

**Published:** 2014-11-19

**Authors:** Huixia Zhao, Nenggan Zheng, Willi A. Ribi, Huoqing Zheng, Lei Xue, Fan Gong, Xiaoxiang Zheng, Fuliang Hu

**Affiliations:** 1 College of Animal Sciences, Zhejiang University, Hangzhou, China; 2 Qiushi Academy for Advanced Studies (QAAS), Zhejiang University, Hangzhou, China; 3 The Private University of Liechtenstein, Dorfstrasse 24, Triesen, Liechtenstein; 4 Department of Biomedical Engineering, Zhejiang University, Hangzhou, China; 5 Key Laboratory of Biomedical Engineering of Ministry of Education, Zhejiang University, Hangzhou, China; University of Sussex, United Kingdom

## Abstract

The insect–machine interface (IMI) is a novel approach developed for man-made air vehicles, which directly controls insect flight by either neuromuscular or neural stimulation. In our previous study of IMI, we induced flight initiation and cessation reproducibly in restrained honeybees (*Apis mellifera* L.) via electrical stimulation of the bilateral optic lobes. To explore the neuromechanism underlying IMI, we applied electrical stimulation to seven subregions of the honeybee brain with the aid of a new method for localizing brain regions. Results showed that the success rate for initiating honeybee flight decreased in the order: α-lobe (or β-lobe), ellipsoid body, lobula, medulla and antennal lobe. Based on a comparison with other neurobiological studies in honeybees, we propose that there is a cluster of descending neurons in the honeybee brain that transmits neural excitation from stimulated brain areas to the thoracic ganglia, leading to flight behavior. This neural circuit may involve the higher-order integration center, the primary visual processing center and the suboesophageal ganglion, which is also associated with a possible learning and memory pathway. By pharmacologically manipulating the electrically stimulated honeybee brain, we have shown that octopamine, rather than dopamine, serotonin and acetylcholine, plays a part in the circuit underlying electrically elicited honeybee flight. Our study presents a new brain stimulation protocol for the honeybee–machine interface and has solved one of the questions with regard to understanding which functional divisions of the insect brain participate in flight control. It will support further studies to uncover the involved neurons inside specific brain areas and to test the hypothesized involvement of a visual learning and memory pathway in IMI flight control.

## Introduction

Insects, due to their impressive flight skills, are ranked among the best models for studying mechanisms of flight control, and for use in the development of biomimetic micro air vehicles (MAVs) [1]. As MAVs have become an increasingly hot topic of research, great advances have been made in the past few decades, but significant challenges still remain with regards to payload mass, flight range, and speed [2], [3]. A novel approach called the insect–machine interface (IMI), which directly controls the flight behavior of insects by either neuromuscular or neural stimulation, has been developed in recent years and promises to overcome some of the challenges facing MAVs [4], [5].

The use of electrical stimulation to induce behavior in insects is not new. Singing behavior of the cricket and grasshopper had been elicited by electrical stimulation of the brain [6], [7] and descending fibres [8], [9]. Rowell (1963) had produced various activities including antennal movements, locomotion, feeding, and sexual behavior in locusts by a chronic electrical stimulation method on different brain sites [10]. And Blondeau (1981) had stimulated neurons in the lobula plate of free-moving and fixed *Calliphora erythrocephala* to evoke course control [11]. In these studies, electrical stimulation was used as one of the tools of neuroethology to investigate the relationship between animal behavior and the nervous system. Beyond that, electrical stimulation was also used to artificially control the locomotion of an autonomous bio-robotic system. Holzer and Shimoyama (1997) had induced the escape turn of cockroach via electrical stimulation to antennae, and built an electronic backpack to control cockroach walking [12].

As to the IMI for MAVs and flight control, researchers managed to elicit flight initiation and cessation in beetles (*Cotinis texana* and *Mecynorhina ugandensis*) by applying electrical stimulation through two electrodes implanted in the bilateral optic lobes [13], [14], [15], [16]. Their experiment was inspired by the immediate flight cessation of untethered *Mecynorhina ugandensis* in response to abrupt darkening of the environment, which led the researchers to hypothesize that the optic lobes might strongly modulate flight initiation and cessation [5]. While alternating positive and negative potential pulses at 100 Hz (20% duty cycle) initiated wing oscillations, a relatively long duration pulse caused wing oscillations to cease [16]. Another study found that stimulating the antennal lobes with 20 Hz, 3.5 V_pp_ pulses initiated flight, while 50 Hz, 3.5 V_pp_ pulses stopped flight, in *Manduca sexta* [17]. In addition, “throttling” (frequency and stroke amplitude of wing oscillation) and turning modulation of insect flight had been achieved by stimulating the optic lobes and basalar muscles [13], [16], neck muscles [17] or ventral nerve cord [18], [19], [20]. One of our previous studies developed a stimulation protocol in restrained honeybees (*Apis mellifera*), using alternating positive and negative electrical pulses (4 V_pp_-40 Hz-40% duty cycle, τ = 5 ms, m = 30) between two microwire electrodes implanted into the bilateral optic lobes to reproducibly generate flight initiation and cessation [21].

It is difficult to elucidate the neuronal mechanisms of IMI from previous studies because identification of these mechanisms requires electrophysiological or optical recordings of neuronal activity in insects during stimulation [16]. We made some preliminary attempts to record neuronal activity, but unfortunately encountered problems due to interference from noise generated by electrical stimulation of the brain (i.e. stimulus artifact) and robust avoidance behaviors of the insects (such as abdomen twitch and wing oscillations). We therefore posed an alternative question, namely how will stimulation of specific brain subregions (with the exception of the optic lobe) affect flight initiation? Solving this question will narrow the search for the neurons involved in IMI to specific brain subregions; by comparing our data with other honeybee neurobiological studies it may be possible to deduce the neural pathways involved.

To accomplish this aim, we first established a new method for localizing brain regions to help with embedding microelectrodes reproducibly into targeted brain subregions. Then, we applied an innovative stimulation protocol to the unilateral brain to study the effects of stimulating different subregions on flight initiation. Lastly, we adopted the method normally used by others to study the effects of neurotransmitters, neuromodulators and neurohormones such as biogenic amines on flight control.

## Materials and Methods

### Animals

Worker honeybees (*Apis mellifera* L.) were captured at the entrance to hives in Zhejiang University and maintained in a perforated bottle containing sugar and wet paper. Throughout the experiments, honeybees were kept warm under a heat lamp (in winter) and regularly supplied with new wet paper.

### Brain microtomy

Paraffin-embedded thick sections: brains were dissected out of head capsules, cleaned to remove fat and tracheae, and fixed in 4% formaldehyde overnight. They were then dehydrated in an increasing ethanol series (50%, 70%, 80%, 2×95%, 2×100%, 15 minutes each), cleared in dimethylbenzene (2×10 minutes), infiltrated with soft wax (2×0.5 h) and hard wax (1 h) before being embedded in hard wax. Consecutive 4 µm thick sections were cut on a rotary microtome (HM 325, MICROM, Germany) using solid razor blades, and were then stained with hematoxylin-eosin before being mounted in neutral balsam.

Araldite-embedded semithin sections: dissected honeybee brains were fixed in a primary fixative containing 2% paraformaldehyde, 2.5% glutaraldehyde and 2.2 g sucrose per 100 ml solution in Millonig's buffer (pH 7.2) for 2–4 h before postfixation in a buffered 2% OsO_4_ solution for 1 h at room temperature. They were then dehydrated in an ethanol series (50%, 70%, 80%, 95%, 10 minutes each and 2×100%, 15 minutes each), cleared twice in propylene oxide (15 and 20 minutes), and placed back into propylene oxide and Araldite mixture (1∶1, 2–3 h) before being embedded in Araldite. Consecutive 1 µm thick sections were cut on a rotatory microtome using a diamond knife and stained with toluidine blue.

### Fixation and positioning system

**Figure 1:**
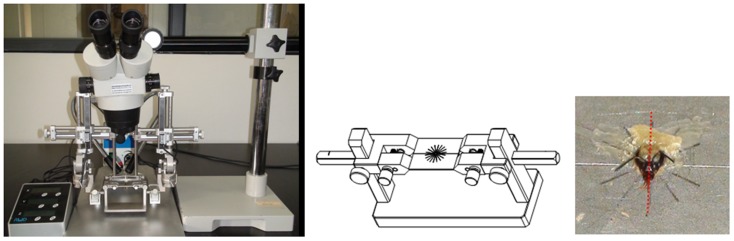
Equipment set-up, honeybee clamping device, and head fixation (the red dotted line indicates head midline).

To assist with positioning the microelectrodes into specific brain subregions, we set up a fixation and positioning system by assembling several pieces of equipment, including a honeybee clamping device (manufactured by our team), a digital stereotaxic apparatus (RWD Life Science Co., China), and a gimbaling stereomicroscope with cold light illuminator (RWD Life Science Co., China). The set-up is shown in Fig. 1.

Honeybees were cold anesthetized before being fixed onto the clamping device. A stereotyped procedure was applied to ensure consistent head position of each fixed honeybee. When the rostral head was pressed down, the interspace between the retral head and the clamp plates was filled up with beeswax–rosin mixture (2∶1). Meanwhile, special attention was paid to adjust the head midline in accordance with a reference line on the clamp plates (Fig. 1). After fixation, the honeybee underwent limb amputation because legs gripping onto the clamp plates would hinder wing movement. Antennae were removed to facilitate electrode implantation. Then, under the stereomicroscope, a rectangular window was cut in the cuticle between two compound eyes and between antennae and ocelli using a scalpel with a carbon steel blade. After removing the glands, membrane and tracheae that cover the brain from the front, intact brain was exposed and perfused with bee saline to keep it moist (Ringer's solution: 130 NaCl, 6 KCl, 4 MgCl_2_, 5 CaCl_2_, 160 sucrose, 25 glucose, 10 HEPES, in mM; pH 6.7, 500 m Osmol).

### Electrode implantation

A new method for locating brain regions was developed to allow implantation of the stimulating electrode into a specific brain subregion. Positional data obtained from frontal and horizontal brain sections were used as references for medio-lateral and antero-posterior (depth) localization of brain subregions respectively. Brain surface landmarks discovered through stereomicroscopy and light microscopy were used as references for dorso-ventral localization.

The stimulating electrodes are Elgiloy–stainless steel microelectrodes, which have Parylene insulation until approximately 66 µm from the tip end (impedance 0.5 MΩ) and 1–2 µm tip diameter (World Precision Instruments, USA). After a stimulating electrode had been embedded in a specific brain subregion, an indifferent electrode made from formvar-insulated nichrome wire (bare diameter 50.8 µm, A–M Systems, USA) was placed in bee saline outside the brain surface to complete the current return path.

### Electrical stimulation

**Figure 2:**
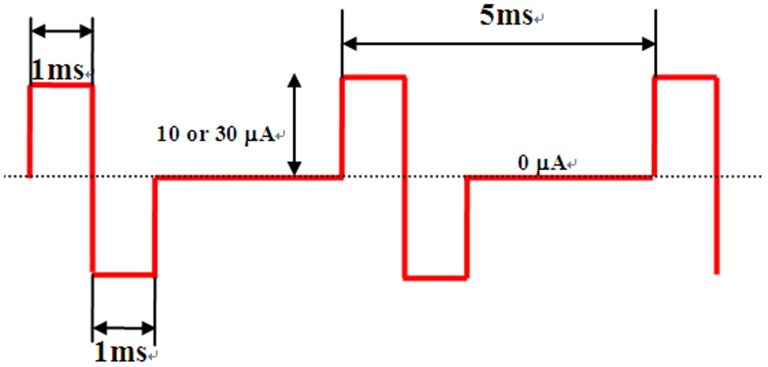
Electrical pulses for stimulation.

A 0.3 s stimulus train of rectangular biphasic (1 ms each phase) pulses at 200 Hz was generated by an Isolated Pulse Stimulator (model 2100, A–M Systems, USA) and used for stimulation. The isolated constant current was set to two amplitudes: 10 µA as low intensity and 30 µA as high intensity (Fig. 2).

Stimulation experiments were carried out in 40 and 100 honeybees using low and high current intensity, respectively. Both experiments were divided equally into two groups in which seven brain subregions were stimulated in opposite sequences to eliminate excitement interactions. Stimulation of each brain subregion was carried out according to the rule that the next stimulus could be given after the most recent stimulus failed to induce flight, and stimulation would be stopped when flight was induced or three stimuli were given. That is consecutive stimuli are used following this rule.

In the pharmacological manipulation experiments, stimulation for all six drug groups was carried out on the α-lobe under low intensity conditions. Videos were made at a specific time point before or after drug delivery (see below Behavior recording), during which honeybee responses to three segregated stimuli were examined.

### Prussian blue verification

After stimulation, the Isolated Pulse Stimulator was modulated to generate a dissociating pulse (20 µA DC, 15–20 s), which partially dissociated Fe^3+^ from the stimulating electrode and deposited it in surrounding brain tissue. Brains were then sectioned in paraffin and dyed with a mixture of potassium ferrocyanide and hydrochloric acid solution to generate prussian blue at the stimulation site.

### Drug dispensing

Three biogenic amines (dopamine hydrochloride, (±)-octopamine hydrochloride, serotonin hydrochloride), acetylcholine chloride, phentolamine hydrochloride (octopamine receptor antagonist) and (+)-butaclamol hydrochloride (dopamine receptor antagonist) (all chemicals were purchased from Sigma-Aldrich Ltd.) were dissolved separately in bee saline at a concentration of 10^−2^ M before they were diluted into 10^−5^ M solutions. For each drug group, 5 µl 10^−5^ M solution was dripped onto the brain surface of ten honeybees using a 50 µl syringe. Electrical stimulation was performed 5 minutes after drug application.

### Behavior recording

A digital camera (HDR-SR12E, Carl Zeiss lens Vario-Sonnar T*, SONY) was positioned in front of the restrained honeybee and used to record behaviors in response to electrical stimulation. In neurotransmitter experiments, three videos were taken at 5-minute intervals before drug delivery, and four videos were taken 5, 10, 15 and 20 minutes after drug delivery.

### Data analysis

The initiation and termination of flight were distinguished by high wing-beat frequency from video frames displayed for 1/25 second using the software Corel VideoStudio Pro X5 (Ulead Information Inc., USA) (see Figure S1).

In the neurotransmitter experiments, flight duration due to each stimulus was calculated from videos. To verify that the honeybee was showing stable performance before application of the drug, we performed pairwise comparisons of flight durations in the three pre-delivery videos. When there were no significant differences, the three pre-delivery videos were considered as an ensemble to compare with each video taken after drug delivery in order to investigate the time-varying effect of drug delivery on flight duration. Lastly, four post-delivery videos were taken as an ensemble to compare with the pre-delivery ensemble. All comparisons were made using non-parametric Mann–Whitney U tests in SPSS statistics 19.0 software. Significant differences were accepted at p<0.05. A group-level analysis was also performed using ANOVA in SPSS.

## Results

### Positional data

Positional data for five brain subregions (α-lobe, ellipsoid body, lobula, medulla, antennal lobe) in two directions, medio-lateral and antero-posterior, were obtained from frontal sections of six brains and horizontal sections of three brains. Two microtomy techniques (see Methods) were used to eliminate methodical error.

**Figure 3:**
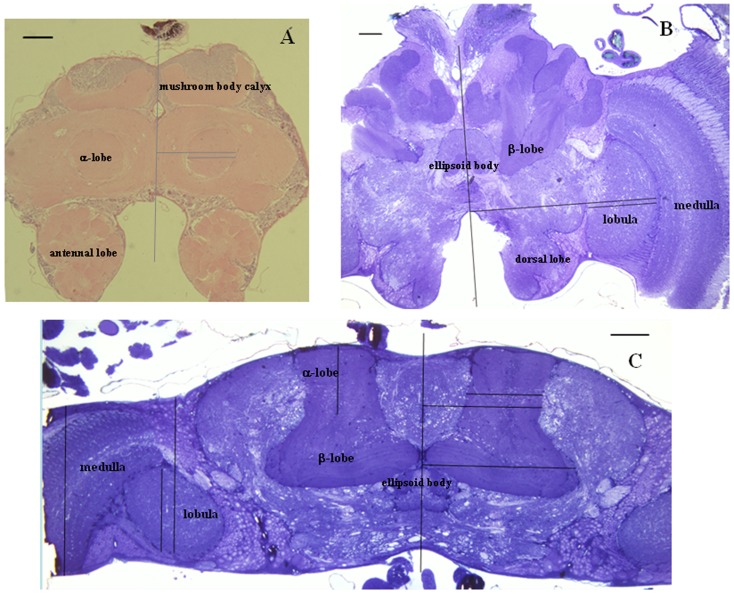
Brain sections and measurement of medio-lateral and antero-posterior positional data. (A) Frontal paraffin thick section showing the α-lobe, mushroom body calyx, and antennal lobe. (B) Frontal Araldite semithin section showing the β-lobe, ellipsoid body, medulla, lobula and dorsal lobe. (C) Horizontal Araldite semithin section showing the α-lobe, β-lobe, ellipsoid body, medulla and lobula. Scale bar  = 100 µm in all pictures.

Eleven slices from serial brain sections of each honeybee were selected and photographed before being measured. The medio-lateral positional data for five brain subregions were measured as perpendicular distance to the brain midline (Fig. 3A, B, C), and antero-posterior positional data were measured as perpendicular depth to the brain surface (Fig. 3C). All data were recorded in Microsoft Excel and processed into curve charts to show the positional distribution of different subregions in the honeybee brain (see Figure S2). Because little is known about the distribution of data for brain subregion positions, we chose the non-parametric Kruskal–Wallis H test to compare the data from different honeybees. Results showed no significant differences in both medio-lateral and antero-posterior positional data for five brain subregions (p>0.05, Table 1). And using non-parametric Mann–Whitney U test to compare the medio-lateral positional data of five brain subregions obtained by two microtomy techniques, we had not found significant differences (p>0.05, Table 2). Mean values for both medio-lateral and antero-posterior positional data were calculated and used to assist electrode implantation (Table 3 and Table 4).

**Table 1:** Comparisons (Kruskal–Wallis H test) of the medio-lateral (N = 6) and antero-posterior (N = 3) positional data obtained from different honeybees.

brain subregions	α-lobe	ellipsoid body	lobula	medulla	antenal lobe
**p value (medio-lateral)**	**0.327**	**0.111**	**0.402**	**0.637**	**0.227**
**p value (antero-posterior)**	**0.618**	**0.149**	**0.088**	**0.096**	**0.165**

**Table 2:** Comparisons (Mann–Whitney U test) between the medio-lateral positional data of five brain subregions obtained by two microtomy techniques.

brain subregions	α-lobe	ellipsoid body	lobula	medulla	antennal lobe
**p value**	**0.347**	**0.561**	**0.119**	**0.261**	**0.098**

**Table 3:** Medio-lateral positional data [µm] for five brain subregions.

compartment	α-lobe	ellipsoid body	lobula	medulla	antennal lobe
**positioning data**	**214 (±93)**	**0 (±70)**	**649 (±116)**	**906(±138)**	**250 (±155)**

**Table 4:** Antero-posterior positional data [µm] for five brain subregions.

compartment	α-lobe	ellipsoid body	lobula	medulla	antennal lobe
**positioning data**	**95 (±95)**	**326 (±47)**	**293 (±111)**	**205 (±205)**	**155 (±155)**

### Discovery of new brain landmarks

**Figure 4:**
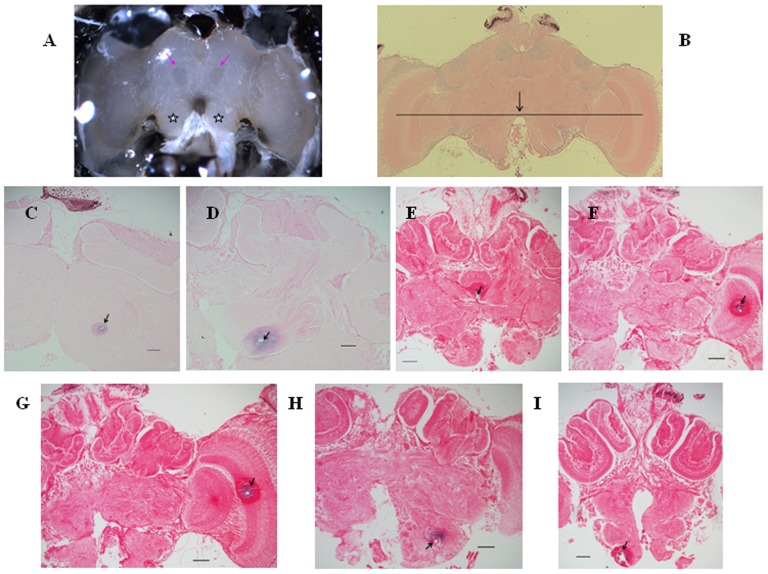
New brain landmarks and prussian blue paraffin sections verifying the stimulation sites. Scale bars 50 µm. (A) Honeybee brain under the stereomicroscope. The two pink arrows indicate landmarks for the α-lobes: small mango-shaped regions. The two stars indicate the antennal lobes. (B) Frontal brain section showing the arc-shaped border of the gap between the two antennal lobes, which is aligned approximately to the centers of the medulla and lobula. (C–I) Paraffin-embedded frontal brain sections dyed with prussian blue. Arrows show the stimulation sites on the α-lobe, β-lobe, ellipsoid body, lobula, medulla, antennal body and suboesophageal ganglion, respectively. Note that each picture shows a hole of around 15 µm with a large surrounding sphere with different staining; this arose because the monophasic dissociating pulse (20 µA DC, 15–20 s) generated polarization and hydrolysis around the electrode tip and produced tissue damage.

We identified some new brain landmarks by stereomicroscopy and light microscopy. Under the stereomicroscope with bilateral cold light illumination, two small mango-shaped regions could be distinguished from their surroundings on the central brain as they were distributed symmetrically along the brain midline and emitted dark aqueous reflection (not obvious under a centralized light source, Fig. 4A). Prussian blue verification of an electrode implanted through the mango-shaped region demonstrated that this area was the α-lobe (Fig. 4C). The two antennal lobes were easily identified under the stereomicroscope from their position ventral to the protocerebrum and dorsal to the antennal roots (Fig. 4A).

Light microscopy of serial horizontal brain sections revealed that the ellipsoid body lies on the brain midline and between the upper borders of the two α-lobes (Fig. 4E). The mango-shaped α-lobe landmarks can thus be used to localize the ellipsoid body too. Microscopy of serial frontal brain sections indicated that the arc-shaped border of the gap between the two antennal lobes was aligned approximately with the centers of the medulla and lobula (Fig. 4B, F, G). The arc-shaped border can thus be used as a landmark for localizing the medulla and lobula. Microscopy of serial frontal brain sections also indicated that the subesophageal ganglion was posterior to the antennal lobes. Prussian blue verification of an electrode implanted deep through the antennal lobe demonstrated that it reached the subesophageal ganglion (Fig. 4H, I). Measurements in serial horizontal brain sections demonstrated that the α-lobe medio-lateral positional data (214±93 µm) was in the range of β-lobe medio-lateral positional data (180±180 µm). Therefore the location of β-lobe could be determined by the α-lobe landmark on surface. Moreover, the α-lobe had a shallow position (95±95 µm) compared to the deep β-lobe (284±85 µm) (Fig. 3C).

### Effects of stimulating different brain subregions on flight initiation

**Figure 5:**
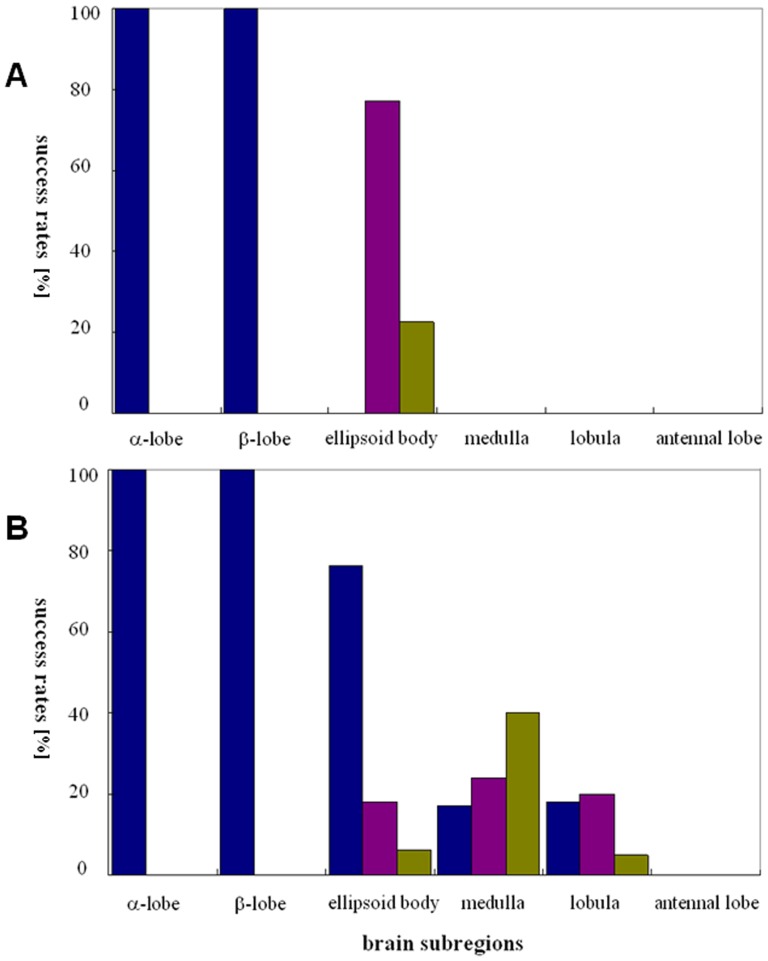
Success rates of flight initiation in response to stimulation of six brain subregions. (A) Low intensity group. (B) High intensity group.

By implanting the electrode through the α-lobe landmark at different depths, we achieved stimulation of either the α-lobe or the β-lobe (Fig. 4C, D). While an electrode implanted at a depth less than 180 µm stimulates the α-lobe, the β-lobe is stimulated by an electrode implanted at a depth of 180–360 µm. Stimulation of the α-lobe consistently triggered flight initiation with a single stimulus in both low and high intensity groups (success rate 100%, N = 40, 100) (Fig. 5A, B). Stimulation of the β-lobe had the same effect on inducing honeybee flight (Fig. 5A, B). We also briefly tested stimulation of the mushroom body calyx, but this failed to induce honeybee flight even with a higher current intensity.

In the low intensity group, however, stimulation of the ellipsoid body required two or three consecutive stimuli to initiate flight in all honeybees (100%, N = 40) (Fig. 5A). But under high current intensity conditions, stimulation of the ellipsoid body was more effective. Of 100 successful cases, 76 honeybees' flight was initiated by one stimulus, and the others by two or three stimuli (Fig. 5B).

Stimulation of the medulla and lobula failed to induce flight initiation in 40 honeybees tested under low current intensity conditions, only inducing lifting of the wings without flapping (Fig. 5A). But when high intensity current was used to stimulate the medulla or the lobula, flight was induced in 43% and 81% of honeybees, respectively (N = 100, Fig. 5B). Of 43 successful cases of medulla stimulation, 18 honeybees initiated flight in response to three consecutive stimuli, 20 with two stimuli and 5 with only one stimulus. For the lobula, successful flight of 17 honeybees were induced by three consecutive stimuli, 24 by two stimuli and 40 by only one stimulus.

Stimulation of the antennal lobe had no effect on flight initiation, but induced folding wings or slight lifting of the wings (Fig. 5A, B). However, stimulation of the deeper subesophageal ganglion (electrode depth >300 µm) with high current intensity successfully induced flight initiation.

To gain a detailed impression of honeybee behaviors in response to stimulation of specific brain subregions, readers are referred to Video S1 and Video S2.

### Effects of biogenic amines or receptor antagonists on flight activity

For six drug groups (60 honeybees in total), flight duration in response to stimulation was tested before drug delivery, and each honeybee was tested nine times, i.e., 540 tests in total. Flight duration varied enormously between individuals (0.23–19.45 s), with an average of 1.8 s. However, comparisons of the nine flight durations recorded from each honeybee did not reveal significant differences (p>0.05, N = 60), which means that all honeybees were showing stable performance before they received a drug.

**Figure 6:**
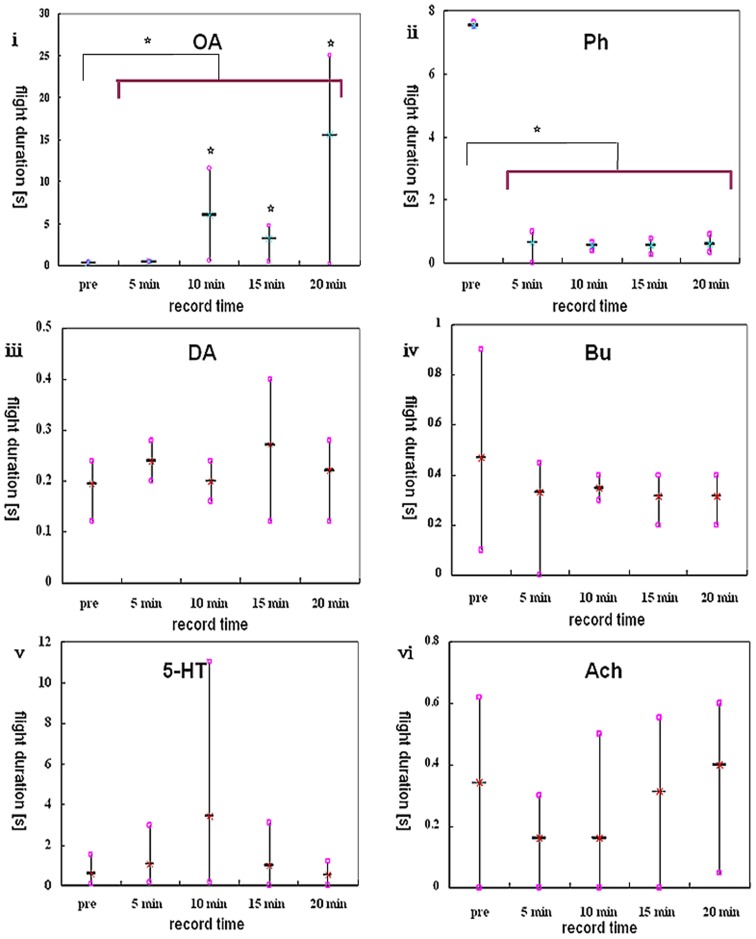
Whisker diagrams showing analysis results. The drug used is abbreviated at the top of each diagram. Stars in **i** indicate that the flight durations from three post-delivery videos are all statistically different from the pre-delivery video ensemble (p<0.05). Two stars above the transverse line in **ii** and **ii** indicate statistical differences in flight durations between two video ensembles (before and after delivery) (p<0.05).

**Figure 7:**
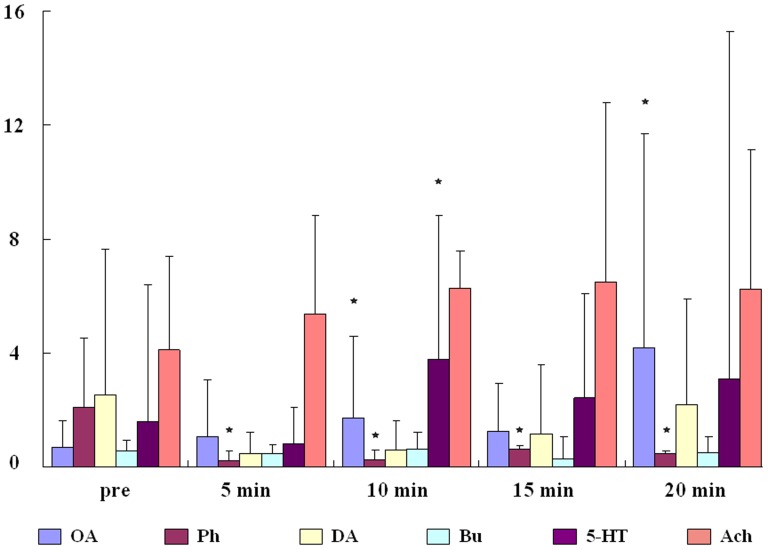
A box and whisker plot showing group-level data analysis, in which the box shows the mean and the whisker shows the standard error (SE). Stars indicate statistical differences that are found in the comparison of group averaged flight durations in pre-delivery ensemble and each post-delivery video by ANOVA.

Whisker diagrams were used to display the analysis results of flight durations tested before and after drug delivery (see Fig. 6). Each diagram shows only one of the ten honeybees tested in a drug group. The mean value of flight duration in the pre-delivery video ensemble or each post-delivery video is shown in the middle as a short horizontal bar with a cross. The higher and lower squares on each vertical line represent the longest and shortest flight durations in that video or video ensemble respectively. Moreover, a box and whisker plot was used to summarize the group averaged data and to show the group differences in flight duration for the different drug types (Fig. 7).

No significant differences were seen in the dopamine (DA), DA-receptor antagonist (butaclamol, Bu), serotonin (5-HT) and acetylcholine (Ach) experiments (p>0.05, N = 40, Fig. 6iii, iv,v,vi). However, significant effects were seen with octopamine (OA) and its receptor antagonist (phentolamine, Ph). Ten honeybees in the OA group all significantly increased their flight duration in response to having 5×10^−11^ mol OA dripped onto their brain surface (p<0.05, N = 10). Comparisons of four single videos obtained after delivery with the pre-delivery video ensemble revealed a delayed flight elongation effect in eight honeybees (Fig. 6i). Significant differences were also obtained when comparing the pre-delivery video ensemble with the post-delivery video ensemble (p<0.05, N = 10, Fig. 6i). By contrast, ten honeybees tested after having 5×10^−11^ mol Ph dripped onto their brain surface all showed significantly shorter flight duration, and indeed flight was sometimes even suppressed completely. Comparisons between the pre-delivery and post-delivery video ensembles revealed significant differences in all honeybees (p<0.05, N = 10, Fig. 6ii), but no significant differences were obtained by comparing the pre-delivery video ensemble with four single post-delivery videos (p>0.05, N = 10).

From group-level analysis, we found statistical differences in both OA and Ph groups which are indicated by stars in Fig. 7. But there was also one star above the box and whisker of 5-HT in the 10 min group. It might be caused by the high variability of flight performance among individuals.

## Discussion

The method of combining brain landmarks with positional data has been proven adequate for the localization of specific brain subregions in honeybees. It was possible to reproducibly localize seven brain subregions. Although this method is not accurate enough to locate the position of specific neurons, it has already allowed great progress to be made in localizing brain regions in vivo. Previous studies have usually used landmarks on the brain surface as references to localize brain subregions, and in combination with recording or stimulation results to localize neurons [22]. For example, the lobula plate in the fly brain can be identified by a characteristic branching pattern of silvery trachea that covers its posterior surface [23]. However, established landmarks for most brain subregions in many model animals (e.g. the honeybee) are still limited. Plus, for many brain subregions, we do not know what the effects of stimulation will be; in fact, these are the problems we wish to solve.

The success rate for flight initiation is higher with electrical stimulation of the unilateral α-lobe (or β-lobe) than with stimulation of the ellipsoid body, which is turn higher than with stimulation of the lobula and the medulla, respectively. Other regions such as the antennal lobe and the protocerebrum area mushroom body calyx (prussian blue verified) failed to induce honeybee flight when stimulated using the same protocol. Tehovnik (1996) stated that the total number of neurons activated directly by a given current was not only dependent on the current intensity but also on the excitability of the neurons [24]. Indeed, when the same brain subregion was stimulated in honeybees in our experiments, the high intensity group (30 µA) always resulted in a higher success rate than the low intensity group (10 µA). Given that electrode placement is not sufficiently accurate to target a specific neuron, we assume that flight initiation is reproducibly induced in different honeybees by the excitation of different groups of neurons within a brain subregion. Accordingly, we have demonstrated that multiple subregions in the insect brain, rather than just the optic lobes, can be used to manipulate flight initiation, but we are not sure if these different subregions have different functions with regards to the exquisite “throttling” and turning modulation of flight.

In their study of beetle flight control, Sato et al. hypothesized two possible neural pathways for the flight initiated by electrical pulses applied between bilateral optic lobes [16]. One possibility is the stimulation directly depolarizes the “giant fiber” motor neurons which connect the insect brain to the flight muscles and mediate the escape flight. Alternatively, the stimulation might depolarize sensory afferents to the brain that lead to alteration of the flight central pattern generator. However, the homologous giant fibers haven't been found in honeybees. And with a number of other armaments to defend themselves, why would honeybees need to escape with such a specialized and hard-wired system? Whereas, studies had found a number of descending neurones (DNs) in honeybees which receive visual inputs from the optic lobes and descend to the thoracic ganglia to control flight course [25], [26]. All of these neurons have their major dendritic arborizations located in the posterior deutocerebrum and immediately lateral to the oesophageal foramen [25], [27]. Some neurons give off branches in the tritocerebrum and suboesophageal ganglion [26]. Our stimulation of the suboesophageal ganglion had induced flight initiation in honeybees. Also, flight was initiated by stimulation of two optic neuropils — the lobula and the medulla, respectively, in some cases. These behavioral responses might realize through the depolarization of such DNs. To make sure the involvement of such DNs in the electrically elicited flight initiation and to make clear how these DNs control the flight muscles, it awaits for the further study of neural recordings.

An interesting potentiation effect of consecutive stimuli was observed in some stimulation experiments of the ellipsoid body and optic lobes. Where a single stimulus could only elicit shock responses (shaking legs, abdomen twitch, sting reflex, lifting wings without flapping), two or three consecutive stimuli induced flight initiation. This effect might have arisen due to the long-term potentiation (LTP). Oleskevich et al. (1997) had reported the first demonstration of long-term synaptic plasticity by long-lasting potentiation in honeybee brain [28]. They recorded the extracellular field response of mushroom body Kenyon cells after electrical stimulation of the olfactory input pathway. LTP was induced by low-frequency stimulation (0.02–1 Hz). Another study discovered the LTP in honeybee brain by electrically stimulating Kenyon cells at high frequency (100 Hz) and recording from a single, identified mushroom body output neuron, the PE1 [29]. Both studies contribute to the mechanism of olfactory learning and memory consolidation. We stimulated the ellipsoid body or optic lobe by consecutive stimuli which have a high frequency (200 Hz) and use a time interval of 2–3 seconds between bouts of stimuli. It seems somewhat likely that our stimulation might induces the LTP in the neural pathway mediating flight initiation.

In addition, we had found another surprising phenomenon when we stimulated the α-lobe first with five or more consecutive stimuli and then stimulated other brain subregions. We found the stimulation of any other brain subregion could induce flight initiation by only one stimulus (personal observation, not included in Results). This phenomenon were held for longer than 10 minutes generally. It is believed that the mushroom bodies of the insect brain are higher-order sensory integration centers that are involved in the memory formation and storage [29]. Together with the potentiation effect we have presented above, it is very likely that our brain stimulation have evoked flight behavior in honeybees through a descending pathway which also associates with a visual learning and memory pathway.

It is generally agreed that octopamine can act as a key neuromodulator as well as a neurohormone to modulate the insect flight [30]. Octopamine can alter the plateau potential and excitability of the interneurons in flight central pattern generator [31], enhance the transmission of neuromuscular junction [32] and the conduction between sensory afferents and motor neurons [33], modulate the responses of proprioceptive sensory neurones [34], promote releasing of the peptidergic adipokinetic hormones [30], and regulate energy metabolism at the onset of flight [35]. But in addition to octopamine, researches also have reported other neurotransmitters, neurohormones or neuromodulators on flight modulation. Claassen and Kammer (1986) have demonstrated that dopamine, octopamine and 5-HT are involved in initiating, maintaining and terminating flight behavior, respectively [36]. Brembs et al. (2007) show that octopamine and tyramine are involved in regulating flight initiation and maintenance through different sites, and therefore exert distinct effects on the flight central pattern generating network [37]. The methods that pressure injecting biogenic amines or antagonists (10^−8^–10^−10^ mol) into thoracic ganglia or superfusing drug solutions directly onto the surface of flight muscles or thoracic ganglia were used as a normal tool by researchers in such studies [36], [38], [39]. Whereas we used an easier way by dripping diluted drug solutions (5×10^−11^ mol) directly onto the brain surface to obtain drug permeation. Though we find this method is not capable of precluding the drug flow into thorax through haemolymph circulation. But from the results, we can conclude that only octopamine (out of octopamine, dopamine, serotonin and acetylcholine) involves in the regulation of electrically elicited honeybee flight. Octopamine applied to the honeybee brain increased flight duration; however, application of an octopamine–receptor antagonist blocked flight induction or reduced flight duration. These results are different from those of others by negating the effect of dopamine and serotonin on flight modulation (see [36]). Perhaps it is because we have induced flight behavior by direct brain stimulation and excited a specific neural circuit, of which a key element is octopamine. But we won't deny the possibility that octopamine might play a role by acting on the central pattern generator (i.e. thoracic ganglia) and flight muscles. Further studies using the picospritzer to limit drugs spreading from the brain to thorax will be done and we will test other drugs such as GABA, histamine and glutamate in modulating the electrically elicited flight behavior.

As a preliminary study, this work has established an experimental system in honeybees and tested the behavioral responses to stimulation of different brain subregions. In conclusion, this work has helped us to understand which functional divisions of the insect brain participate in flight control, and will support further studies to uncover the involved neurons from specific brain areas via a neurophysiological approach, and also to test the hypothesis regarding of the possible memory processing.

## Supporting Information

Figure S1 **Screenshots of two adjacent video frames displayed in 1/25 second by software Corel VideoStudio Pro X5.** Honeybee flight initiates in the second screenshot which is distinguished by the high wing-beat frequency.
(TIF)

Figure S2 **A curve chart displays the medio-lateral positioning data of five brain subregions obtained from one honeybee brain frontal sections.** Numbers on horizontal coordinate represent the measured brain slices from front to back. And the vertical coordinate shows the positioning data. Curves in the color of green, purple, dark blue, orange and light blue represent the brain subregion of ellipsoid body, α-lobe, antennal lobe, lobula and medulla respectively. Triangles on each curve represent the subregion centers and verticle lines indicate the subregion's extended diameter.
(TIF)

Video S1 **Induced flight by one stimulus on α-lobe in restrained honeybee.**
(AVI)

Video S2 **Induced flight by three stimuli on medulla in restrained honeybee.**
(AVI)
