# Supplementary figures and images for: Neuromechanism Study of Insect–Machine Interface: Flight Control by Neural Electrical Stimulation
Source: PLoS One. 2014 Nov 19;9(11):e113012. doi: 10.1371/journal.pone.0113012 (PMC4237392; doi:10.1371/journal.pone.0113012)

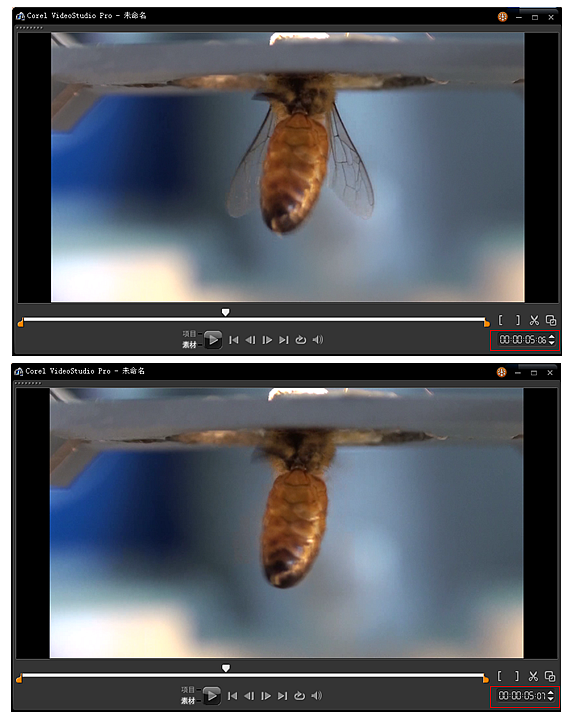

Supplement: Figure S1 — Screenshots of two adjacent video frames displayed in 1/25 second by software Corel VideoStudio Pro X5. Honeybee flight initiates in the second screenshot which is distinguished by the high wing-beat frequency. (TIF) [file pone.0113012.s001.tif]

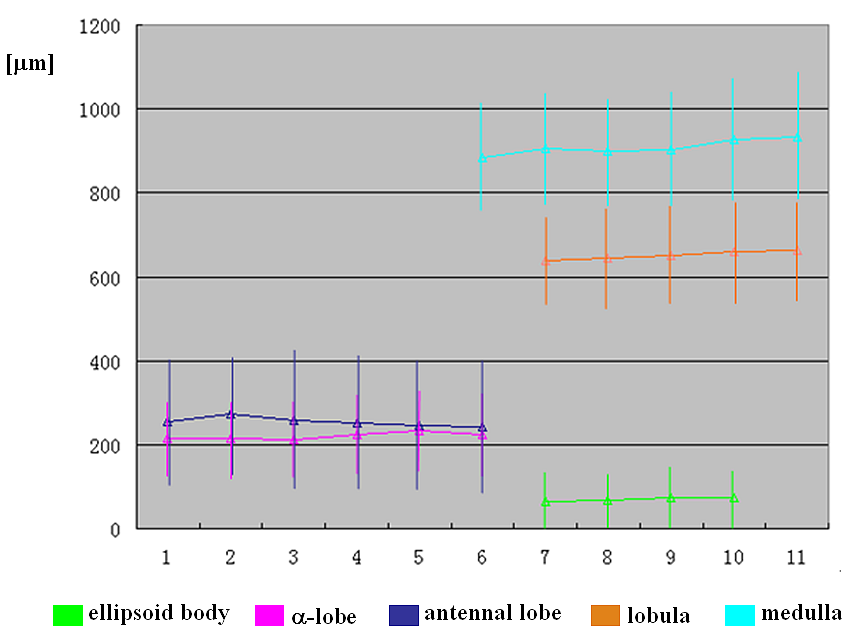

Supplement: Figure S2 — A curve chart displays the medio-lateral positioning data of five brain subregions obtained from one honeybee brain frontal sections. Numbers on horizontal coordinate represent the measured brain slices from front to back. And the vertical coordinate shows the positioning data. Curves in the color of green, purple, dark blue, orange and light blue represent the brain subregion of ellipsoid body, α-lobe, antennal lobe, lobula and medulla respectively. Triangles on each curve represent the subregion centers and verticle lines indicate the subregion's extended diameter. (TIF) [file pone.0113012.s002.tif]
